# miR-140-5p and miR-140-3p: Key Actors in Aging-Related Diseases?

**DOI:** 10.3390/ijms231911439

**Published:** 2022-09-28

**Authors:** Léa Toury, Diane Frankel, Coraline Airault, Frédérique Magdinier, Patrice Roll, Elise Kaspi

**Affiliations:** 1 Aix Marseille Univ, INSERM, MMG, 13005 Marseille, France; 2 Aix Marseille Univ, APHM, INSERM, MMG, Hôpital la Timone, Service de Biologie Cellulaire, 13005 Marseille, France

**Keywords:** microRNA, miR-140, aging-related diseases

## Abstract

microRNAs (miRNAs) are small single strand non-coding RNAs and powerful gene expression regulators. They mainly bind to the 3′UTR sequence of targeted mRNA, leading to their degradation or translation inhibition. *miR-140* gene encodes the pre-miR-140 that generates the two mature miRNAs miR-140-5p and miR-140-3p. miR-140-5p/-3p have been associated with the development and progression of cancers, but also non-neoplastic diseases. In aging-related diseases, miR-140-5p and miR-140-3p expressions are modulated. The seric levels of these two miRNAs are used as circulating biomarkers and may represent predictive tools. They are also considered key actors in the pathophysiology of aging-related diseases. miR-140-5p/-3p repress targets regulating cell proliferation, apoptosis, senescence, and inflammation. This work focuses on the roles of miR-140-3p and miR-140-5p in aging-related diseases, details their regulation (i.e., by long non-coding RNA), and reviews the molecular targets of theses miRNAs involved in aging pathophysiology.

## 1. Introduction

The first microRNA (miRNA), lin-4, was described in 1993 in the nematode *Caenorhabditis elegans*, by the Ambros and Ruvkun groups [1,2]. Since then, more than 48,800 mature miRNAs have been discovered from 271 organisms; among them, 2654 mature miRNAs sequences have been reported in humans (miRBase v.22) [3,4].

miRNAs are small non-coding RNAs involved in gene expression regulation. The average length of miRNAs is 22 nucleotides (nt), of which seven correspond to the seed region (nucleotides 2 to 8). Genes encoding microRNAs are intergenic or intragenic. In the first case, a specific promoter regulates transcription; in the second case, miRNA genes are mainly located in introns, whereas few of them are located in exons [5,6,7]. 

Conventionally, miRNAs genes are transcribed to primary miRNAs (pri-miRNAs; >1 kb) using RNA polymerase II. Then, pri-miRNA is processed by a complex including DiGeorge Syndrome Critical Region 8 protein (DGCR8) and a RNase III enzyme Drosha, which cleaves the pri-miRNA into a 70 nt stem-loop precursor miRNA (pre-miRNA) [8,9,10,11,12]. Pre-miRNA exportation to the cytoplasm is controlled by RanGTP/exportin 5 complex [13,14,15]. Lastly, the cytoplasmic RNase III endonuclease Dicer removes the terminal loop, leading to a ≈ 25 nt miRNA duplex [16,17]. This duplex is integrated in “RNA-induced silencing complex”, called “RISC-complex”, containing the Argonaute protein (AGO). The passenger strand is eliminated from the duplex, whereas the guide strand, corresponding to the mature (≈22 nt) miRNA, is conserved [18]. Both the 5p and 3p strands, arising from the 5′ or 3′ end of the duplex, respectively, can be the guide strand, depending to different factors; e.g., the cell type, developmental stage, enrichment of purines at the 5′end, and miRNA thermodynamic stability [19].

Several non-classical miRNA biogenesis pathways have also been described, independent of Drosha, Dicer, or AGO proteins [5]. Mirtrons are a class of intronic but non-canonical miRNAs that are processed by a DGCR8/Drosha-independent pathway and are generated by spliceosome from direct intron splicing. “Debranched” mirtrons are similar to canonical pre-miRNAs and are exported by Exportin-5 in the cytoplasm. Their further maturation is identical to the canonical manner [20,21,22].

Intronic miRNAs genes are embedded into a host gene and are mostly processed according to the protein-encoding gene in which they are hosted. In a few cases, intronic miRNA expression differs from the expression of the host gene: miRNA transcription can be independent from the host gene promoter, as the miRNA genes can be regulated by their own independent promoter [23,24,25,26].

MiRNAs mainly regulate gene expression due to messenger RNA (mRNA) interactions, inside the RISC complex: the seed region located to the 5′ end of microRNAs usually targets the 3′ untranslated transcript region (3′UTR) of mRNA, leading to mRNA degradation or translation inhibition, according to the base complementarity between these two sequences. In a few cases, a miRNA targets the 5′UTR of mRNA or coding sequence, and this interaction has been reported to induce translation under specific conditions (e.g., in quiescent cells) [27,28,29,30]. It has also been well established that some miRNAs can be reimported into the nucleus, where they directly interact with gene promoters, thus inducing gene transcription [31,32].

Considering all these mechanisms, miRNAs are considered major actors in gene expression regulation. In addition, one microRNA is rarely specific to one mRNA, and one mRNA can be targeted by different microRNAs sharing the same seed region. Many studies and reviews are available reporting miRNA involvement in physiological pathways and developmental processes, as well as their deregulation under pathological conditions leading to diseases, such as cancers or genetic diseases. They also have been proposed as potential biomarkers or therapeutic targets in various pathological contexts [33,34,35,36,37].

## 2. miR-140-5p and miR-140-3p Biogenesis

**Figure 1:**
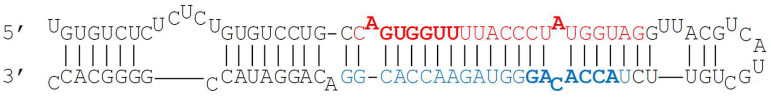
Pre-miR-140 sequence. Representation of the pre-microRNA-140 sequence obtained and modified from miRBase (https://www.mirbase.org/cgi-bin/mirna_entry.pl?acc=MI0000456, accessed on 20 August 2022). The mature sequences of miR-140-5p and miR-140-3p are highlighted in red and blue, respectively. The seed regions of each miRNA are in bold.

Gene encoding microRNA-140-5p/-3p is located on chromosome 16q22.1, and is hosted in the 15th intron of the *WW domain containing E3 Ubiquitin protein ligase 2 (WWP2)* human gene. In mice, *miR-140* gene is localized in the 16th intron of *WWP2* gene [7,38]. Its transcription generates two transcripts, miR-140-3p and miR-140-5p, processed from the same pre-miR. *miR-140* gene transcription is independent of the transcription of its host gene, and is regulated by transcription factors, such as Nuclear factor of activated T-cells 3 (NFAT3) and Mothers against decapentaplegic homolog 3 (SMAD3) [38]. Both miR-140-3p and miR-140-5p can be the guide strand and silence mRNAs. Interestingly, as these two miRNAs do not share the same seed region (Figure 1), the predicted targeted mRNAs are thus different [38]. miR-140 (i.e., miR-140-5p in the former studies) was first described as cartilage specific [39]. A large number of studies have demonstrated its key role in osteoarthritis (OA) development. Both miR-140-5p and miR140-3p have also been proposed as plasmatic biomarkers for several diseases and their involvement in cancers has largely been described [40]. In cancer, as well as in an aging context, miR-140-5p and miR-140-3p target molecules involved in cell senescence: anti-senescent molecule forkhead box Q1 (FOXQ1) [41] is a target of miR-140-3p in bladder carcinoma [42], and miR-140-5p enhances cell senescence due to Peptidyl-prolyl cis-trans isomerase NIMA-interacting 1 (PIN1) repression, in a model of progenitor cell aging [43].

This review focuses on the roles of miR-140-3p and miR-140-5p in neurodegenerative diseases, atherosclerosis, visual acuity loss, osteoporosis, and osteoarthritis, which are all age-related diseases, and details the molecular targets of these miRNAs involved in aging pathophysiology.

## 3. Role of miR-140-5p/-3p in Neurodegenerative Diseases/Cognitive Impairment

Neurodegenerative diseases, such as Alzheimer’s disease (AD), Parkinson’s disease, or post-stroke infarction, lead to cognitive impairment with aging. Several miRNAs have recently been considered as neuroregulators, as well as neuroprotectors, leading to their consideration as biomarkers or therapeutic targets for neuronal cell death [44].

To our knowledge, no study has evidenced miR-140-5p/-3p involvement in Parkinson’s disease. In contrast, two recent publications have highlighted the neurotoxic role of miR-140-5p in AD, based on the study of different AD models. AD is caused by accumulation of β-amyloid (Aβ) in the brain, forming neurotoxic amyloid plaques. miR-140-5p overexpression was observed in hippocampal tissues from a rat model of AD, and associated with neurological function impairment. In hippocampal mouse neurons from an in vitro model of AD, miR-140-5p overexpression (using mimics transfection) led to reactive oxygen species (ROS) production and mitochondrial dysfunction, by directly targeting *phosphatase and tensin homolog-induced putative kinase 1 (PINK1)*, involved in mitochondrial function preservation [45]. miR-140-5p is also upregulated in post mortem brains from AD patients, compared to cognitively normal controls, causing a deleterious circle as it directly targets *A Disintegrin and Metalloproteinase 10 (ADAM10)* and *SRY-box transcription factor 2 (SOX2),* a transcription factor that positively regulates ADAM10 transcription. ADAM10 expression is thus reduced in AD, leading to Aβ plaque accumulation [46].

Plasmatic miR-140-5p/-3p has also been proposed as a predictive tool for cognitive function evaluation during aging: miR-140-5p levels are positively associated with cognitive performance measures [47]. miR-140-3p, in combination with other defined miRNAs, could represent a plasmatic biomarker, predictive for mild cognitive impairment (MCI—an intermediate state between normal aging and dementia) in elderly populations [48].

Similarly, the miR-140-5p plasmatic level is enhanced during acute ischemic stroke in humans [49]. Interestingly, in a mouse model of ischemic stroke (middle cerebral artery occlusion), miR-140-5p expression was drastically decreased in response to ischemic stroke, in comparison to control mice; while miR-140-5p ectopic overexpression in brain prevented neuronal apoptosis and restrains the development of ischemic injury in this model, via toll-like receptor 4/nuclear factor-kappa B (TLR4/NF-κB) axis regulation [50]. Similar results were obtained in a rat model of ischemia stroke [51,52]. miR-140-3p was also described as neuroprotective from cerebral ischemia-reperfusion injury in an in vitro model (PC12 cells) [53]. Moreover, miR-140-5p directly targets the 3’UTR of *Vascular Endothelial Growth Factor A (VEGFA)*, a pro-angiogenic factor and inhibits endothelial cell properties in vitro (proliferation, migration, and pseudo-capillar formation) under normoxic and hypoxic conditions [52].

These studies suggest that miR-140-5p is downregulated after ischemic stroke. Preventing the downregulation of miR-140-5p and, to a lesser extent, miR-140-3p could be neuroprotective and represent a new therapeutic approach, by inhibiting neuro-inflammation and abrogating angiogenesis following ischemic brain injury.

## 4. Role of miR-140-5p/-3p in Atherosclerosis

Atherosclerosis (AS) is one of the main factors leading to cardiovascular diseases, which are the leading cause of death worldwide [54]. AS is characterized by formation of a plaque in the arterial intima, essentially composed of lipids, such as triglycerides and oxidized Low-Density Lipoprotein (ox-LDL), generating immune and inflammatory responses. Ultimately, plaque growth or plaque rupture causes damage as it induces vessel obstruction [55,56]. In atherosclerotic plaques, phagocytosis is activated by ox-LDL; macrophages accumulate lipids and derive into foam cells. Ox-LDL are the origin of a vicious circle, as they stimulate ROS production, thus accentuating ox-LDL generation. They cause cellular damage in macrophages and they stimulate macrophages phagocytosis and activation into foam cells [57]. During AS, vascular smooth muscle cell (VSMC) dysfunction was also evidenced. Phenotypic alterations, such as gain of proliferation and migration, contribute to plaque progression and vascular injury [58].

Opposite results were published concerning the role of miR-140-5p in lipid accumulation into macrophages-derived foam cells: in a cellular model of AS macrophages (ox-LDL treated THP1 cells), miR-140-5p expression was inversely correlated with ox-LDL macrophages content, suggesting that in AS, miR-140-5p is downregulated. miR-140-5p could participate as a brake in AS, by targeting TLR4. Indeed, miR-140-5p mimic transfection in AS macrophages prevented lipids accumulation, ROS production, and cell apoptosis [57].

In contrast, Zhao and colleagues demonstrated, in similar in vitro model, that miR-140-5p expression was enhanced in ox-LDL-treated macrophages. They showed that miR-140-5p silences Regulatory Factor X7 (RFX7), a transcription factor that binds to the promoter of *ABCA1* gene to induce its expression [59]. This gene encodes ATP-binding cassette transporter A1 (ABCA1), an ABC transporter involved in cholesterol efflux outside macrophages [60]. miR-140-5p could thus amplify the lipid accumulation in atherosclerotic plaque macrophages, participating to AS aggravation. Other arguments support these results. In ApoE^−/−^ mice, an in vivo model of AS, overexpression of miR-140-5p accelerated plaque formation in aorta, in association with reduced levels of RFX7 and ABCA1 in this tissue [59].

miR-140-5p is also involved in VSMC alterations during AS. In AS aorta from ApoE^−/−^ mice, miR-140-5p was enhanced compared to wild type mice, thus increasing oxidative stress, resulting from the increase of ROS production and the decrease of antioxidant molecules (superoxide dismutase (SOD) and gluthathione (GSH)) [61]. Sirtuin 2 (SIRT2) and nuclear factor erythroid 2-related factor 2 (NRF2), two direct targets of miR-140-5p, may strongly participate in oxidative stress in AS. The mechanistic role of SIRT2 in oxidative stress remains unclear as SIRT2 regulates hypoxia inducible factor 1 (HIF1) expression, but its positive regulation (stabilization) or negative regulation (degradation) is still debated [61,62]. In response to oxidative conditions, NRF2 is translocated into the nucleus, where it acts as a transcription factor activating anti-oxidative gene transcription [61,63]. miR-140-5p expression was also significantly higher in artery tissues from AS patients versus healthy controls, as well as in ox-LDL-treated human VSMCs compared to control conditions. In addition, miR-140-5p inhibition represses AS VSMC migration, proliferation, and stimulates apoptosis, via rescue of roundabout guidance receptor 4 (ROBO4) expression, a vascular-specific receptor involved in angiogenesis [64].

LDL-cholesterol clearance from blood circulation also contributes to reducing atherosclerotic plaque formation. Plasmatic LDL-cholesterol endocytosis, via LDL-receptors (LDLR) on hepatocytes, is a major event controlling plasmatic LDL-cholesterol levels. In humans, the seed region of miR-140-5p was predicted to hybridize with 3′UTR of *LDLR*. This prediction was experimentally confirmed in human hepatic cells, in which miR-140-5p inhibits the cell surface LDLR expression and reduces LDL-cholesterol absorption, while simvastatine reverts this phenomenon, as this drug is able to reduce miR-140-5p expression [65]. miR-140-5p expression seems to be endogenously regulated during AS. Prostate cancer antigen 3 (PCA3), a long non-coding RNA (lncRNA), physiologically sponges miR-140-5p in macrophage-derived foam cells, thus repressing its deleterious role in AS [59]. Moreover, the plasmatic level of miR-140-5p is inversely correlated with the lncRNA metastasis-associated lung adenocarcinoma transcript 1 (MALAT1), which may inhibit miR-140-5p [66].

Concerning miR-140-3p, this miRNA was significantly downregulated in AS aorta from ApoE^−/−^ mice, leading to MKK6 and TP53RK proteins overexpression, compared to control aorta. These two kinases have been reported to be involved in signaling cascades playing a pivotal role in endothelial injury [67,68]. Similarly, in a commercial cell line of human coronary endothelial cells (HCAECs), miR-140-3p directly targets *mitogen-activated protein kinase 1 (MAPK1)*, encoding a serine/threonine protein kinase that belongs to the MAPK family. Activation of MAP kinase signaling results in the production of inflammatory cytokines and chemokines via NF-kB nuclear importation [69,70].

In a vascular injury model (i.e., in-stent restenosis), miR-140-3p was downregulated in arterial smooth muscle cells (ASMC) isolated from restenosis artery wall compared to normal arteries, resulting in abnormal ASMC proliferation enhancement and apoptosis inhibition, which contributed to arterial damage. In this model, *C-Myb* and *B-cell lymphoma 2 (BCL-2)* are no longer targeted by miR-140-3p, causing their upregulation. C-Myb is a transcription factor known to induce ASMC proliferation, while BCL-2 is an outer membrane mitochondrial anti-apoptotic protein [71].

As described for miR-140-5p (see above), several non-coding RNAs are predicted or proven to sponge miR-140-3p in AS. In plasma from coronary atherosclerotic heart disease patients, miR-140-3p was inversely correlated with the lncRNA nuclear enriched abundant transcript 1 (NEAT1) [69]; and in AS aorta from ApoE^−/−^ mice, miR-140-3p was negatively regulated by circRNA_36781, also called circRNA_ABCA1 [67], leading to miR-140-3p downregulation and facilitating endothelial injury in these models.

## 5. miR-140-5p/-3p and Visual Acuity Loss

During aging, visual acuity loss occurs. Age-related macular degeneration (AMD) is the leading cause of visual impairment in Europe [72]. AMD is divided into two clinical entities: wet and dry AMD [73]. In a cohort of 33 wet AMD patients versus 31 controls, the expression of a panel of 384 microRNAs was investigated in plasma. In this study, miR-140-3p, among 10 others, was found to be downregulated in the AMD group, suggesting that plasmatic miRNAs levels could be predictive for wet AMD. Nevertheless, this observation needs to be confirmed in a larger cohort, and no molecular mechanisms were explored, to highlight the potential role of miR-140-3p in wet age-related macular degeneration [74].

In age-related cataract, another cause of visual acuity loss occurring during aging, no studies have shown a role for miR-140-5p/-3p, unlike other miRNAs [75].

## 6. Role of miR-140-5p/-3p in Osteoporosis

Osteoporosis is a systemic bone metabolic disorder, characterized by dysregulation of bone turnover, associated with low bone density and mass and accelerated bone loss, leading to bone fragility and fractures [76]. Osteoporosis affects life quality during aging, notably in postmenopausal women. To prevent this worldwide health problem, regular physical exercise is an effective method for stimulating bone osteogenesis in osteoporotic patients, and which improves bone density and reduces osteoporosis-induced risk of fracture [77].

Osteogenic differentiation was induced in a model of bone marrow mesenchymal stem cells (BMSCs) submitted to tensile strain, a mechanical stimulation mimicking physical exercise at the cellular level, together with lncRNA-MEG3 expression enhancement; the latter represses miR-140-5p expression through a “sponging” mechanism [78]. Nevertheless, in a cohort of idiopathic osteoporosis patients with prevalent low-traumatic fractures (pre- and postmenopausal women, and men), seric miR-140-5p, among other miRNAs, was downregulated in comparison to healthy subjects and was inversely correlated to body mass index (BMI) [79].

Conversely, an elevated seric level of miR-140-3p could be a biomarker of osteoporosis, osteopenia, and fractures in post-menopausal women, as miR-140-3p was overexpressed in the serum of osteoporotic post-menopausal women, compared to healthy post-menopausal women [80]. These results were reinforced by Yin et al., who reported higher levels of miR-140-3p in PBMC and serum from osteoporotic post-menopausal women compared to healthy controls. Moreover, in an osteoblastic progenitor cell line (C2C12 cells), miR-140-3p silencing induced cell proliferation and differentiation, and inhibited apoptosis, by targeting Phosphatase and TENsin homolog (PTEN) and thus, inactivating the Phosphatase and TENsin homolog/ Phosphoinositide 3-kinase/ serine/threonine kinase 1 (PTEN/PI3K/AKT) signaling pathway [81]. These results corroborate an older publication, which highlighted the overexpression of miR-140-3p in BMSCs from a model of osteoporotic rats, compared to control animals. The inhibition of miR-140-3p in this cell model promoted osteogenesis [82].

The role of miR-140-3p in osteoblastic differentiation has still not been totally elucidated, as opposite results have been reported [83]: Fushimi et al. demonstrated that miR-140-3p overexpression activates osteoblasts differentiation by targeting Transforming Growth Factor-β3 (TGF-β3) [84]. In contrast, Mao et al. showed in MC3T3-E1, an osteoblastic cell line obtained from mouse calvarium, that upregulation of miR-140-3p suppresses viability and differentiation, by targeting MCF.2 cell line derived transforming sequence-like (MCF2L) [85].

## 7. Role of miR-140-5p/-3p in Osteoarthritis

Osteoarthritis (OA) is a chronic degenerative joint disease that affects quality of life in elderly populations. OA is associated with articular pain and stiffness, leading to functional disabilities. Several predisposing factors have been identified such as age, gender, obesity, and traumatic injury. Extracellular matrix (ECM) remodeling is impaired, in response to chondrocytes senescence and lesser proteoglycan secretion by chondrocytes. High levels of proinflammatory cytokines (e.g., Interleukin-6 (IL-6)) and matrix-degrading enzymes (e.g., Matrix metallopeptidase 13 (MMP13) and a disintegrin and metalloproteinase metallopeptidase with thrombospondin type 1 motif 5 (ADAMTS5)) are secreted in OA cartilage and synovial fluid. The elevated levels of reactive oxygen species (ROS) associated with oxidative stress cause oxidative damage, leading to OA [86]. During aging, various epigenetic changes occur, such as DNA methylation, histone modifications, and miRNAs regulation. Theses mechanisms are also present in aging-related OA.

miR-140-3p and miR-140-5p are physiologically expressed in cartilage and chondrocytes, and have been highlighted as key actors in OA [87,88]. In normal conditions, miR-140-5p expression increases during chondrogenesis, in parallel to chondrogenic differentiation markers (SRY-related high-mobility group box 9 (SOX9) and Collagen type-II (COL2A1)), as the levels are higher in mature chondrocytes than in Mesenchymal stem cells (MSCs) [89]. Mouse models were generated to investigate the miR-140-5p/3p involvement in cartilage development and regeneration. Specific deletion of the *miR-140* gene sequence in the intronic region of *Wwp2* gene led to the abolishment of both miR140-3p and miR-140-5p expression in these miR-140^−/−^ mice, whereas transgenic mice overexpressing both miR140-3p and miR-140-5p in cartilage were generated, using *pri-miR-140* insertion near the cartilage-specific *Col2a1* promoter [90]. Mouse developmental observations showed that miR-140^−/−^ mice exhibited a mild skeletal phenotype with a short stature, and during aging, OA damage was associated with ECM degradation and cartilage fibrosis. Moreover, transgenic mice were protected from OA development in a model of antigen-induced arthritis [90]. miR-140-5p was reduced in joint tissues from old mice compared to healthy young mice [91] and many teams have described a downregulation of miR-140-5p expression in human OA-cartilage, in comparison to healthy cartilage [38,89,92]. This decrease of miR-140-5p in OA-cartilage was corroborated by in vitro models of OA-chondrocytes, which have also objectified miR-140-5p expression decreases [89,93,94].

Regulatory mechanisms for miR-140-5p are opposed, independently of those regulating WWP2: NFAT3 increases miR-140-5p levels in OA-chondrocytes, and SOX9 physiologically stimulates miR-140-5p expression during chondrogenesis [38,95]. Conversely, several mechanisms negatively regulate miR-140-5p expression in OA-chondrocytes: TGF-β/SMAD3, hypermethylation of CpG sites of the regulatory region of *miR-140* gene, and Interleukin-1β (IL-1β) stimulation [38,88,96]. Moreover, in chondrocytes extracted from OA cartilage tissue, LINC01534 was overexpressed in comparison to healthy cartilage tissue, and this lncRNA directly targets and sponges miR-140-5p. LINC01534 overexpression is mediated by IL-1β [97].

**Figure 2:**
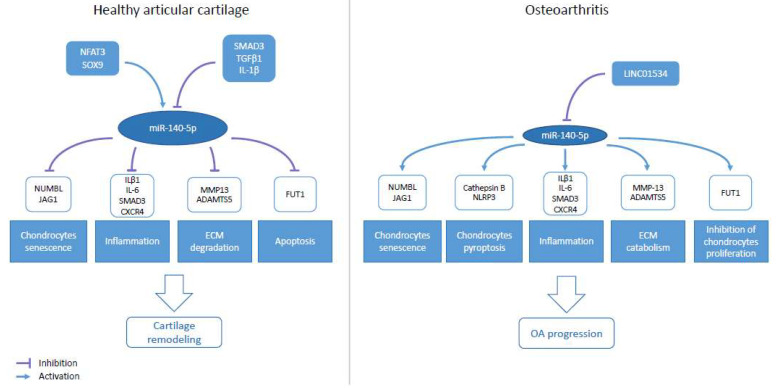
Role of miR-140-5p in osteoarthritis (OA). Schematic representation of miR-140-5p regulators and targets, and biological consequences, in cartilage remodeling and OA progression.

In an in vitro model of OA chondrocytes (IL-1β treatment), miR-140-5p was decreased compared to non-stimulated chondrocytes. In this model, miR-140-5p transfection prevented chondrocytes senescence, and ECM degradation, as the expression of two major ECM components (COL2 (collagen type II) and ACAN (Aggrecan)), was restored, in association with a reduction of ECM degradation enzymes [89]. Comparatively, in a rat OA model, intra-articular injection (IAJ) of miR-140-5p attenuates OA progression and prevents chondrocyte senescence [93,94,98]. During OA, miR-140-5p downregulation contributes to cartilage remodeling impairment. The absence of inhibition of miR-140-5p targets in chondrocytes results in chondrocytes senescence (via NUMB-like endocytic adaptator protein (NUMBL) and Jagged1 (JAG1) in the Notch pathway), chondrocytes pyroptosis (apoptosis occurring in an inflammatory state) (via cathepsin B/Nod-like receptor protein 3), inflammation (via IL-1β, Interleukin-6 (IL-6), SMAD3, C-X-C motif chemokine receptor 4 (CXCR4)), ECM catabolism (MMP13, ADAMTS5), and inhibition of chondrocytes proliferation (fucosyltransferase 1 (FUT1)) (Figure 2) [88,90,99,100,101,102,103,104,105].

miR-140-3p is also expressed in cartilage to a greater extent than miR-140-5p [87]. Similarly to miR-140-5p, miR-140-3p is downregulated in OA-chondrocytes [38]. This downregulation may be the result of direct targeting of miR-140-3p by the lncRNA LINC01385 in OA-tissues [106]. A few targets of miR-140-3p have been identified as being involved in OA progression: miR-140-3p attenuates OA progression and regulates chondrogenesis due to CXCR4 and Ras-like proto-oncogene (RALA) inhibition, respectively [99,100]. miR-140-3p level has been monitored in the serum from OA patients before and after high tibial osteotomy (HTO), which represents one option for OA-treatment, as cartilage regeneration is expected after this surgery. miR-140-3p expression is significantly up-regulated 6 and 18 months post-surgery, indicating that the miR-140-3p seric level may represent a prognostic biomarker for the cartilage repair process [107].

Considering all these data, miR-140-5p/-3p represent serious targets for OA treatment. As described previously, intra-articular injection of miR-140-5p in animal models of OA slows down OA progression [93,94,98]. To protect miR-140-5p from degradation, one team generated exosomes overexpressing miR-140-5p (miR-140-5p transfected cells) or not (untransfected cells) and derived from human synovial mesenchymal stem cells. These overexpressing miR-140-5p exosomes stimulated chondrocytes proliferation in vitro and prevented OA progression in a rat model [108]. In humans, gene editing has been performed using the CRISPR/Cas9 method, in order to silence miR-140-5p/-3p expression in primary OA chondrocytes, without *WWP2* expression modulation. This recent development has only been used at this stage to identify miR-140-5p/-3p targets, but opens up great possibilities as a tool to further evaluate the role of miRNAs in AO and to assess potential new therapies [109].

## 8. Conclusions

miR-140-5p/-3p have been widely studied in the context of cancer, but also in diseases related to aging, particularly in osteoarthritis. Both miR-140-5p and miR-140-3p are derived from the same gene, but have different targets. Despite this, these two miRNAs are often involved in the same pathologies with similar or opposite actions. They are key actors in the pathophysiology of many diseases and/or can be used as predictive circulating biomarkers.

A list of miRNAs regulating aging-related pathways, such as senescence, DNA damage response, has been established; the miRNAs involved in physiological aging are thus, grouped under the term “geromiRs” [110]. Even if miR-140-5p/-3p are not listed as “GeromiRs”, this review highlights that miR-140-5p/3p, and particularly miR-140-5p, are involved in the most common aging-related pathologies. Considering the major involvement of miR-140-5p/-3p in physiological aging, it would be interesting to analyze the role of these miRNAs in the pathophysiology of diseases with accelerated and premature aging.
